# The NUTRAOLEOUM Study, a randomized controlled trial, for achieving nutritional added value for olive oils

**DOI:** 10.1186/s12906-016-1376-6

**Published:** 2016-10-22

**Authors:** Sara Biel, Maria-Dolores Mesa, Rafael de la Torre, Juan-Antonio Espejo, Jose-Ramón Fernández-Navarro, Montserrat Fitó, Estefanía Sánchez-Rodriguez, Carmen Rosa, Rosa Marchal, Juan de Dios Alche, Manuela Expósito, Manuel Brenes, Beatriz Gandul, Miguel Angel Calleja, María-Isabel Covas

**Affiliations:** 1 Fundación Pública Andaluza para la Investigación Biosanitaria de Andalucía Oriental “Alejandro Otero” (FIBAO), Granada, Spain; 2 Instituto de Nutrición y Tecnología de Alimentos “José Mataix” (INyTA), Granada, Spain; 3 Integrative Pharmacology and Neurosciences Systems Research Group and Cardiovascular Risk and Nutrition Research Group. Hospital del Mar Research Institute (IMIM), Barcelona, Spain; 4 Spanish Biomedical ResearchNetworking Centre, Physiopathology of Obesity and Nutrition (CIBERobn), Instituto de Salud Carlos III, Madrid, Spain; 5 Universitat Pompeu Fabra, (CEXS-UPF), Barcelona, Spain; 6 Instituto para la Calidad y Seguridad Alimentaria (ICSA), Granada, Spain; 7 CM EUROPA, Jaen, Spain; 8 Biologia Reproductiva de Plantas. Consejo Superior de Investigaciones Científicas (CSIC), Granada, Spain; 9 Antimicrobianos Naturales. Biotecnología de los Alimentos. Instituto de la Grasa, Consejo Superior de Investigaciones Científicas (CSIC), Sevilla, Spain; 10 Química y Bioquímica de Pigmentos. Fitoquímica de los Alimentos. Instituto de la Grasa. Consejo Superior de Investigaciones Científicas (CSIC), Sevilla, Spain; 11 NUPROAS Handelsbolag, Nackã, Sweden; 12 NUPROAS HB, Spanish Office: Apartado de Correos 93, 17242 Girona, Spain

**Keywords:** Olive oil, Virgin olive oil, Functional olive oil, Olive oil polyphenols, Maslinic acid, Oleanolic acid

## Abstract

**Background:**

Virgin olive oil, a recognized healthy food, cannot be consumed in great quantities. We aim to assess in humans whether an optimized virgin olive oil with high phenolic content (OVOO, 429 mg/Kg) and a functional one (FOO), both rich in phenolic compounds (429 mg/Kg) and triterpenic acids (389 mg/kg), could provide health benefits additional to those supplied a by a standard virgin olive oil (VOO).

**Methods/design:**

A randomized, double-blind, crossover, controlled study will be conducted. Healthy volunteers (aged 20 to 50) will be randomized into one of three groups of daily raw olive oil consumption: VOO, OVOO, and FOO (30 mL/d). Olive oils will be administered over 3-week periods preceded by 2-week washout ones. The main outcomes will be markers of lipid and DNA oxidation, inflammation, and vascular damage. A bioavailability and dose-response study will be nested within this sustained- consumption one. It will be made up of 18 volunteers and be performed at two stages after a single dose of each olive oil. Endothelial function and nitric oxide will be assessed at baseline and at 4 h and 6 h after olive oil single dose ingestion.

**Discussion:**

For the first time the NUTRAOLEUM Study will provide first level evidence on the health benefits *in vivo* in humans of olive oil triterpenes (oleanolic and maslinic acid) in addition to their bioavailability and disposition.

**Trial registration:**

The Trial has been registered in ClinicalTrials.gov ID: NCT02520739.

## Background

In human randomized and controlled studies olive oil, particularly the virgin one, rich in phenolic compounds, has been shown to provide benefits on key processes for atherosclerosis development such as oxidative damage, inflammation, cell adhesion molecules, and endothelial dysfunction. In addition, a protection for hard primary endpoints such as atrial fibrillation and diabetes by virgin olive oil consumption has also been reported [1].

Olive oil (OO), besides its high content of a healthy fat, the monounsaturated oleic acid, has minor components with bioactive properties classified as: 1) the unsaponifiable fraction, which is extracted with solvents after OO saponification, and 2) the soluble fraction which includes the phenolic compounds [1]. The content of the phenolic compounds (also known as polyphenols, when they are the complex ones) of an OO depends of the cultivar, climate, olive variety and ripeness, and the type of processing. Virgin olive oil (VOO) obtained from the first press or centrifugation of the olives has the highest phenolic content [1]. On November 2011, the European Food Safety Authority (EFSA) released a claim concerning the benefits of the daily ingestion of OOs rich in phenolic compounds on low-density lipoproteins (LDL) oxidation [2], a known factor for coronary heart disease development. The use of the claim in OO bottles, is regulated in the Commission Regulation (EU) N° 432/2012 [3] as: “The claim may be used only for OO which contains at least 5 mg of hydroxytyrosol and its derivatives (e.g. oleuropein complex and tyrosol) per 20 g of olive oil. This implies that only high phenolic content OOs can bear the claim. Due to this, the need to optimize the OO processing in order to obtain high phenolic content OOs is one of the current goals in terms of increasing the nutritional value of an olive oil.

Among minor OO components triterpenic acids from the OO unsaponifiable fraction have also potential benefits for health. The seeds and the skin of the olives, used to produce pomace olive oil, are very rich in triterpenic acids. Pomace olive oil and oleanolic and maslinic acids, have shown to have bioactive properties in cellular and animal models [4–6]. The human health benefits of triterpenes from olive oil have not been at present evaluated. We hypothesized that an optimized VOO with a high phenolic content (OVOO) and a functional olive oil (FOO) rich in both phenolic compounds and triterpenic acids can provide additional benefits, to those provided by an standard VOO, on risk factors for coronary heart disease (CHD) in humans. We are also aimed to assess the bioavailability and disposition of oleanolic and maslinic acids in humans, a key point for supporting the rationale of their health benefits.

According to the Evidence Based Medicine [7], the healthy properties of OVOO and FOO must be tested in proper clinical randomized trials, versus VOO obtained by traditional procedures, for ensuring that their benefits for health are really highlighted.

## Method/design

### Aim of the study

The objectives of the present trial are on one hand to assess the benefits of the enrichment of VOO with both olive oil polyphenols and triterpenes on lipid and DNA oxidation, inflammation, and endothelial function in healthy volunteers. On the other hand, the study is aimed to assess the bioavailability of oleanolic and maslinic acids from olive oil in humans.

The primary null hypothesis is: The efficacy for protecting against lipid and DNA oxidation, inflammation, and endothelial dysfunction of the olive oils will be as follows: FVOO > OVOO > VOO.

In order to be able to obtain future health claims from EFSA or FDA for the functional OOs we will also examine the bioavailability in humans of their active principles (phenolic compounds and triterpenes such as oleanolic and maslinic acids), and its time-and-season variability. Our primary null hypothesis is that the biovailability of triterpenes in humans will be dose-dependent of the triterpenic content of the olive oils.

### Characteristics of the olive oils

Olive oils will be specially prepared for the trial from an extra virgin olive oil (VOO), produced from Picual olives (Andalucía, Spain). They will have similar fat and micronutrient, (i.e vitamin E, sitosterols, etc..) composition with exception of their phenolic/triterpene content. A VOO obtained by a traditional procedure will be used as a control. An optimized virgin olive oil (OVOO) will be obtained by mixing the best olive oils from several olive varieties with very high phenolic content and by using optimized extraction procedures. A functional olive oil (FOO), rich in both high phenolic compounds and triterpenes, will be obtained by addition to the OVOO of an extract of oleanolic and maslinic acids obtained from olive skin by physical procedures. Characteristics of the olive oils to be used in the study (pilot data) are shown in Table 1.

**Table 1:** Characteristics of the olive oils to be used in the NUTRAOLEUM Study

Component	VOO	OVOO	FOO
Free acidity (% of oleic acid)	0.13	0.12	0.13
Peroxide value, (meq O2/kg oil)	6.9	7.7	9.1
K270	0.11	0.12	0.12
K232	1.42	1.35	1.43
Esqualene (mg/100 g)	529	536	545
Fatty acids (%)			
Palmitic	10.0	10.4	10.2
Palmitoleic	0.4	0.6	0.6
Estearic	2.3	2.2	2.1
Oleic	78.9	78.2	78.4
Linoleic	6.6	6.8	6.9
Linolenic	0.6	0.7	0.7
Arachidonic	0.4	0.4	0.4
Eicosanoic	0.3	0.4	0.4
Total phenolic compounds (ppm)	124	490	487
Hydroxytyrosol and derivates	105	424	423
Lignanes	18.2	61.3	59.2
Flavonoids	0.7	3.4	3.2
Simple phenols	0.0	0.9	0.9
Triterpenic acids (mg/kg)	86.3	86.5	389
Maslínic	47.3	47.3	218
Oleanolic	39.2	39.1	171
Sterols (ppm)	1437	1396	1460
Campesterol (%)	2.9	3.0	3.0
Stigmasterol (%)	0.7	0.7	0.8
B-Sitosterol (%)	94.2	94.2	94.6
D7-Stigmastanol(%)	0.3	0.4	0.3
Eritrodiol + Uvaol (%)	1.5	1.5	1.4
Alpha-tocopherol	183	174	176
Total carotenoids (ppm)	7.1	6.8	7.0
Beta-carotene	2.6	2.3	2.7
Lutein	3.1	3.4	3.1
Xantophyll pigments	1.3	1.1	1.2
Chlrophyll pigments	8.7	10.8	9.8
Clrophyll a + b	0.4	0.5	0.5
Pheophytyn a + b	7.7	9.6	8.8

*VOO* virgin olive oil, *OVOO* optimized virgin olive oil with a high phenolic content, *FOO* funcional olive oil rich in phenolic compounds and oleanolic and maslinic acids

### Design

The study is a randomized, double-blind, crossover, controlled, and double-center trial. The trial was conducted in Virgen de las Nieves and San Cecilio General Hospitals of Granada, Spain. The trial comprises a sustained consumption study and, nested within, a dose-response study in which the bioavailability of active principles, phenolic compounds and triterpenes will be assessed. After 6-months, the bioavailability study will be repeated in order to assess time-and-season variations in the bioavailability of the active principles. The study will be sequentially conducted as follows: enrollment after screening via inclusion and exclusion criteria, randomization, treatment periods, and assessment.

**Fig. 1:**
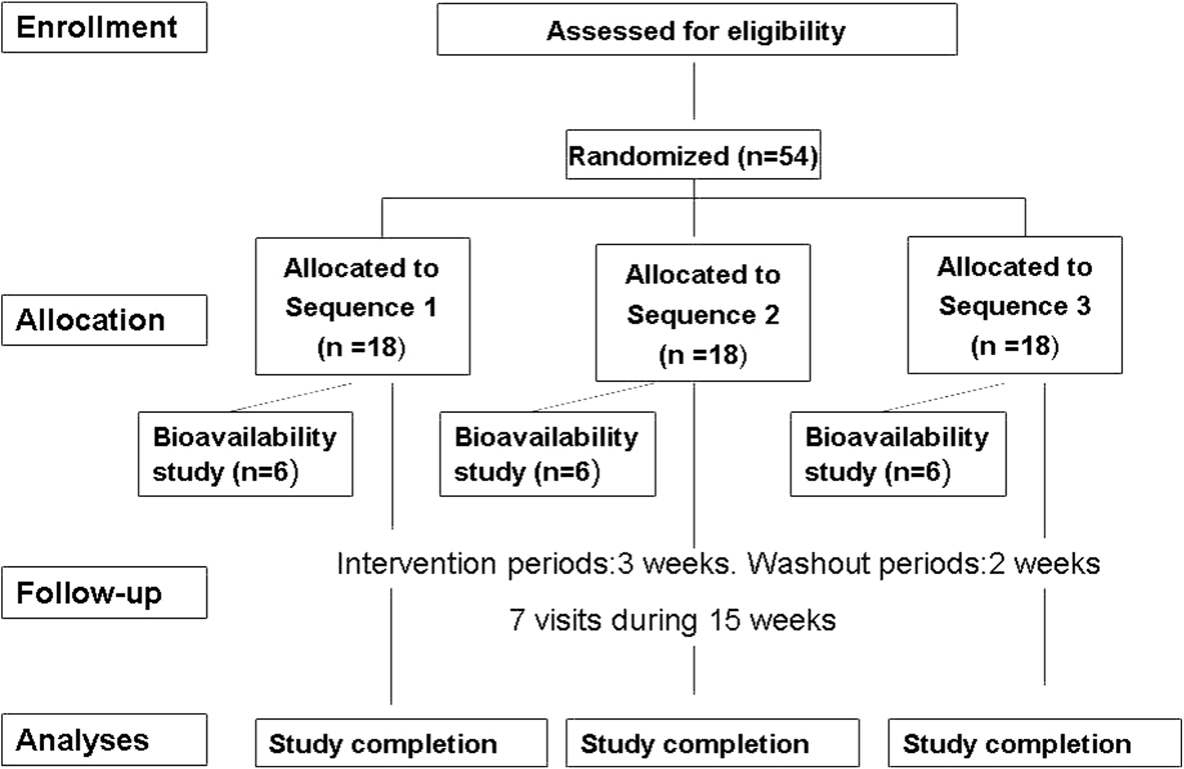
Flow chart of the study. Sequence 1: **a**, **b**, and **c** olive oils; Sequence 2 :**b**,**c**, and **a** olive oils; Sequence 3: **c**, **a**, and **b** olive oils

The protocol will be conducted in accordance with the Declaration of Helsinki and the Good Clinical Practice Guidelines [8]. All investigators are appropriately qualified to conduct and supervise the trial. All patients will provide written informed consent prior to study entry. The flow chart of this study is shown in Fig. 1.

### Participants and eligibility

#### Inclusion criteria

Individuals aged from 20 to 60, healthy on the basis of physical examination and routine biochemical and hematological laboratory determinations, with willingness to provide written informed consent and to adhere to the protocol.

#### Exclusion criteria

Smoking, intake of antioxidant supplements, aspirin, or any other drug with established antioxidant properties, hyperlipidemia, obesity (body mass index >30 kg/m^2^), diabetes, hypertension, celiac or other intestinal disease, any condition limiting mobility, life-threatening diseases, or any other disease or condition that would impair compliance.

### Recruitment

Participants will be recruited from the general population of Granada, Spain, by using newspaper and tableaux advertisements in civic centers. A total of fifty-four individuals (27 males and 27 women) are expected to be recruited in this trial.

### Randomization and allocation concealment

Using a stratified block randomization method, the eligible patients will be randomly allocated to three sequences of olive oil administration. Random numbers will be generated by a computerized random-number generator using the block-randomization method of a software program for sequence generation by an independent statistician. The randomization lists will be concealed in a lightproof sealed envelope. The sealed envelopes will be kept by the independent statistician of the study. Participants, investigators, and outcome assessors will be blinded to the treatment allocation throughout the course of the study.

### Blinding and code breaking

Olive oils will be coded, providing masking to the treatment assignment to ensure double blinding. Participants, clinicians, technicians, outcome assessors, and investigators will be unaware of the sequence group assignments or block size. If a participant has a serious adverse event and an immediate cessation of trial treatment is required, the blinding code will be broken. Daily doses of olive oil will be prepared in special containers with the three types of olive oil labeled “A”,“B”, and “C”. Containers with the corresponding 30 mL olive oil daily dose will be delivered at the beginning of each intervention period to the participants.

### Intervention

Participants will be randomly assigned to three sequences of olive oil administration. Olive oils (VOO, OVOO, and FOO) will be sequentially administered over three periods of 3 weeks preceded by 2-week washout periods in which participants will be requested to avoid olives and olive oil consumption. During olive oil intervention periods participants will be requested to ingest a raw daily dose of 30 mL of olive oil distributed over three meals. During washout periods participants will be provided of sunflower olive oil for raw and cooking purposes. Details of the sustained consumption study procedures are shown in Table 2. For participants entering in the dose-response bioavailability study at the beginning of each intervention period the corresponding 30 mL daily dose of olive oil (VOO, OVOO, or FOO) will be administered as a single dose at fasting state. After 6 months the bioavailability study will be repeated to assess time-and-season variations. Details of the bioavailability study procedures are shown in Table 3.

**Table 2:** Detailed study procedures in the sustained consumption study (0 mL/day, 3-week intervention periods)

	Study Period							
Visit (V) number *WO, washout period* *IP, Intervention period*	Enrollment	V1 *(WO*)	V2 *(IP 1)*	V3 *(WO*)	V4 *(IP 2*)	V5 *(WO*)	V6 *(IP 3*)	V7 *(Close out*)
Time Point, week (Week range)	Up to -12	0 (0–2)	2 (2–5)	5 (5–7)	7 (7–10)	10 (10–12)	12 (12–15)	15
Screening routine laboratory analyses	X							
Demographic/ habits information-taking	X							
Informed consent form	X							
Randomization & allocation	X							
Treatment distribution		X		X		X		
Blood and 24 h-urine collection at fasting		X	X	X	X	X	X	X
Assessments								
*General outcomes*								
General medical questionnaire		X	X	X	X	X	X	X
Alcohol consumption questionnaire		X		X		X		X
3-day dietary record		X		X		X		X
Minnesota Leisure Time Physical Activity questionnaire		X						X
Blood pressure and anthropometric		X	X	X	X	X	X	X
Visual Analogue Scale				X		X		X
Monitoring adverse effects				X		X		X
Compliance check (containers)				X		X		X
*Primary outcomes*								
Plasma Lipid profile		X	X	X	X	X	X	X
Plasma Lipid and DNA oxidation		X	X	X	X	X	X	X
Plasma Inflammation markers		X	X	X	X	X	X	X
*Secondary outcomes*								
Plasma Antioxidant vitamins		X	X	X	X	X	X	X
Plasma fatty acids		X	X	X	X	X	X	X
Serum Endothelin		X	X	X	X	X	X	X
Ex vivo cell immune response		X		X		X		X
*Compliance markers*								
Urinary tyrosol and hydroxytyrosol		X	X	X	X	X	X	X
Plasma Triterpenes		X	X	X	X	X	X	X

**Table 3:** Detailed study procedures in the bioavailability study (30 mL single dose)

Visit (V) number *IP, Intervention period*	Enrolment	V2 *(IP 1)*	V4 *(IP 2*)	V6 *(IP 3*)
Time Point, week	Up to -12	2	7	12
Informed consent form	X			
Randomization & allocation	X			
Treatment distribution		X	X	X
Blood collection at baseline (0 h) and at 30 min, 45 min, 1 h, 2 h, 4 h, 6 h, 8 h, 10 h, 12 h, and 24 h after oils ingestion		X	X	X
Urine collection at baseline (0 h) and at 0–2 h, 2–4 h, 4–6 h, 6–8 h, 8–10 h, 10–12 h, 12–24 h, after oils ingestion		X	X	X
Assessments				
*Primary outcomes*				
Urinary tyrosol and hydroxytyrosol at 0–2 h, 2–4 h, 4–6 h, 6–8 h, 8–10 h, 10–12 h, 12–24 h, after oils ingestion		X	X	X
Plasma Triterpenes at baseline (0 h) and at 30 min, 45 min, 1 h, 2 h, 4 h, 6 h, 8 h, 10 h, 12 h, and 24 h after oils ingestion		X	X	X
Endothelial function measurements at baseline (0 h) and at 4 h, and 6 h, after oils ingestion		X	X	X
*Secondary outcomes*				
Plasma gastrointestinal hormones t baseline (0 h) and at 2 h, 4 h, and 6 h, after oils ingestion		X	X	X

### Dietary control

A three-day dietary record will be registered at the beginning of the study and after each intervention period. Energy consumption and dietary intakes of macro- and micronutrients will be calculated from the 3-day records using an appropriate software. To avoid an excessive intake of antioxidants and phenolic compounds during the clinical trial period, participants will limit the consumption of the followings foods to: 2 servings of vegetables or pulses/day; 3 servings of fruits (including juices)/day; 3 cups of tea or coffee/day; 1 piece of a bar of chocolate (15 g)/day; 2 glasses of wine/day; 30 g/week of nuts; fish, with preference for the white one, to a maximum of 2 servings (150 g/serving)/week; to avoid products canned with oils, such as sardines, peppers, etc…Participants will be asked to avoid to take meals out of home, if possible, and to bring with them the individual olive oil containers for their use at meals. Participants will be personally advised by a nutritionist on how to record food consumption and follow the above mentioned dietary recommendations.

In the dose-response bioavailability study, after an overnight fast, volunteers will ingest 30 mL of one of the three olive oils with a standard piece of bread (80 g). At 6 h and at 10 h after the olive oil ingestion participants will receive a low-phenolic content meal or snack avoiding the previously described foods. During the previous 3-days of each intervention period, participants were requested to avoid moderate or intense physical activity.

### Outcome measures

In the sustained consumption study we will assess outcome measures at the beginning of the study, and before and at the end of each olive oil intervention period (Table 2). Biological samples will be collected at fasting state of minimum 10 h, and 24-h urine will be also collected. In the dose-response bioavailability study blood samples and urine collection will be collected at several times at postprandial time (Table 3). Serum and plasma will be obtained by centrifugation of blood at 1500 g at 4 °C for 20 min and stored at − 80 °C in the central laboratory’s biobank of Virgen de las Nieves Hospital. Whole blood will be collected at baseline and at 5 h after olive oil intake in PAXgene tubes for future gene expression analyses and stored at −80 °C after 2 h at room temperature.

#### Primary outcomes

Primary outcomes will be markers of lipid and DNA oxidation, inflammation, and endothelial function. Concerning DNA oxidative damage, there is evidence of its role in various diseases, particularly in cancer, but also in atherosclerosis. Among DNA oxidative damage biomarkers, urinary 8-oxo-7,8-dihydro-20-deoxyguanosine (8-oxo-dG) has received particular interest [9]. A recent meta-analysis has established that 8-oxo-dG levels are higher in patients with CVD than in controls [10]. DNA repair is thought to be a major contributor to urinary 8-oxo-dG levels, diet, per se, and cell turnover playing negligible roles [11]. However, reactive oxygen species (ROS) induced 8-oxo-dG can promote DNA hypomethylation by inhibiting DNA methylation at cytosine bases located nearby. This ROS-induced DNA hypomethylation is related to both malignant transformation and tumor progression [12]. In our experience [13–15] and that from others [16], consumption of antioxidant-rich foods, including olive oil, and dietary patterns, such as the Mediterranean diet, have shown to decrease urinary 8-oxo-dG concentrations in healthy individuals and metabolic syndrome patients. Due to this, we selected to assess DNA oxidative damage, through 8-oxo-dG measurement, as a primary outcome.

At the beginning of the study, and before and at the end of each olive oil intervention period, serum glucose, cholesterol, and triglycerides will be determined by standard enzymatic methods in a PENTRA-400 autoanalyzer (ABX-Horiba Diagnostics, Montpellier, France). HDL cholesterol will be measured as a soluble HDL cholesterol determined by an accelerator selective detergent method (ABX-Horiba Diagnostics,Montpellier, France). LDL cholesterol will be calculated by the Friedewald formula. Oxidized LDL will be determined in serum by a sandwich ELISA procedure using the murine monoclonal antibody mAB- 4E6 as capture antibody, and a peroxidase conjugated antibody against oxidized ApoB bound to the solid phase (ox-LDL, Mercodia AB, Uppsala, Sweden).). Conjugated dienes in LDL will be measured spectrophotometrically at 234 nm after Cu^++^ oxidation. Urine total F_2α_-isoprostanes (8-iso-PGF_2α_) and 8-oxo-dG will be determined by ELISA (Oxford Biomedical Research, Michigan, USA, and JaICA, Japan Institute for the Control of Ageing, Fukuroi, Shizuoka, Japan, respectively). Specific biomarkers of inflammation and cardiovascular risk, including adiponectin, resistin, myeloperoxidase (MPO), plasminogen activator inhibitor-1 (PAI-1), tumour necrosis factor-alpha (TNF-α), interleukin-6 (IL-6), interleukin-8 (IL-8), soluble intercellular adhesion molecule-1 (sICAM-1) and soluble vascular adhesion molecule-1 (sVCAM-1), will be assessed by Luminex 200 system with the XMap technology (Luminex Corporation, Austin, TX, USA) using human monoclonal antibodies (Milliplex Map Kit, Millipore, Billerica, MA, USA. Within the frame of the bioavailability study endothelial function will be measured by peripheral arterial tonometry with the EndoPAT 2000 device (Itamar Medical Inc., Caesarea, Israel), at baseline and at 4 and 6 h after the ingestion of the single 30 mL dose of the olive oils (Table 3).

#### Secondary outcomes

Systolic and diastolic blood pressures will be measured with a mercury sphygmomanometer after a minimum of 10 min rest in the seated position; the average of two measurements will be recorded. Physical activity, a potential confounder variable concerning oxidative and anti-inflammatory status will be recorded at the beginning and at the end of the study, and assessed by the Minnesota Leisure Time Physical Activity Questionnaire validated for its use in Spanish men and women [17, 18]. A visual analogue scales (VAS) for measurement of appetite sensations will be administered at the end of each intervention period [19].

Tocopherol, beta-carotene, retinol, and Coenzymes Q_9_ and Q_10_ will be measured by high-pressure liquid chromatography (HPLC) coupled to mass spectrometry detection (HPLC-MS) with after extraction with 1-propanol. Plasma fatty acids will be measured after methylation by gas-liquid chromatography coupled to mass spectrometry detection (GS-MS). Serum endothelin will be determined by ELISA (R&D Systems, Minnesota, USA). After whole blood *ex vivo* stimulation cultures, with challenging molecules such as E.coli lipopolysacaride or a Phaseolus Vulgaris phytohemaglutinin, an additional panel of immune biomarkers: IL-1 beta, IL-2, IL-4 and interferon gamma, among others will be measured by Luminex. Within the frame of the bioavailability studies, nitric oxide (spectrometry), and gastrointestinal hormones (grelihn, amilin, gastric inhibitory polypeptide (GIP), glucagon-like peptide-1 (GLP-1), pancreatic polypeptide (PP), polypeptide YY (PYY) and colecistokinine will be performed at baseline and at 2, 4, and 6 h after the ingestion of a single dose of the olive oils (Table 3).

#### Compliance markers

Tyrosol and hydroxytyrosol in urine samples [20], and oleanolic and maslinic in plasma, will be determined by gas chromatography-mass spectrometry (GC/MS) at the beginning of the study and before and after intervention periods in the sustained consumption study. Postprandial kinetics of the biomarkers will be measured at the bioavailability study (Table 3).

### Withdrawal, dropout, discontinuation and compliance

Participants will be allowed to withdraw at any time during this clinical trial. Participants who withdraw will be followed to investigate the reason for withdrawal. Participants may be advised to discontinue this trial if there is a serious intervention related adverse event or if the participant is noncompliant with the study procedure. Participants will be instructed to return the individual olive oil containers when collecting the next daily doses for the amount of unconsumed olive oil to be registered. Participants whose compliance with treatments or placebo is ≤ 80 % of the total will be considered to have dropped out.

### Standard operation procedures for quality assurance

We have established detailed standard operating procedures for this clinical trial and have educated all practitioners, nurses, and technicians and set qualification standards to make sure the patients are treated with high standards and in accordance with the trial protocol. Practitioners and medical personnel will attend a 2-day training workshop about the trial procedures, and be aware of any change in the protocol. A written protocol and standardized recording documents will be provided. All personnel involved in the study, will be blinded to group allocations. A First Entry Data Monitoring and a Field Control Quality Visit at the middle of the study will be performed by external audit (NUPROAS HB) which is independent from field work investigators and the sponsor.

### Adverse events and safety monitoring

Although the treatments are natural foods, olive oils, all unexpected adverse events related to the interventions will be reported to the investigators by participants and written on the individual case report form by the investigator. Each participant will be monitored for adverse events (pain at acne lesions or other sites, nausea/vomiting, fatigue, allergic reaction, and any adverse events related to intervention) after each visit.

### Sample size and power analysis

Accepting an alpha risk of 0.05 and a beta risk of 0.20 in a two-sided test, at least 40 subjects in the sustained consumption study are necessary to recognize as statistically significant a difference greater than or equal to 10 units in the oxidized LDL. A dropout rate of 15 % was anticipated. This sample size will also permit to show, as statistically significant with a power of 80 %, a difference of 10 lag-time units among treatments in the clinical trial. Drop-outs before the first intervention period will be replaced in order to avoid drop-outs greater than 15 %. We will enlarge the sample size to 54 in order to have more statistical power for our results.

### Statistical analyses

Normality of variables will be assessed by normal probability plots. One-factor ANOVA or Kruskal-Wallis test will be used to determine differences in basal characteristics and nutrient intake among the three olive oil interventions as appropriate. Period and sequence effects concerning a carryover effect will be assessed by means of an Analysis of Variance for repeated measures. The possible carryover effect will be corrected by the adjustment of final values after intervention periods by the values at the corresponding baseline period for each intervention. A general linear model for repeated measurements, with Tukey’s correction for multiple comparisons, will be fitted in order to assess differences between values at the beginning and at the end of each intervention period, and at postprandial state. Linear regression models will be used to adjust values at the end of the intervention periods for basal values in order to assess the linear trend across interventions. The paired comparison of post-intervention target concentrations will be carried out by a General Linear Mixed Model (GLMM) with the olive oil type (VOO, OHPCO, FOO) as fixed factor; individual level of test subjects as random effect; and, basal values for each intervention period, olive oil administration order, and age. Statistical significance was defined as *P* < 0.05 for a two-sided test. These statistical analyses will be performed using IBM SPSS Statistics 19.

## Discussion

The NUTRAOLEUM Study is designed to assess whether, besides an increase in the polyphenol content of the olive oil (OVOO), and additional enrichment of this OVOO with oleanolic and maslinic acids from olive seeds (Functional OO, FOO) will increase the healthy benefits of these OOs versus an standard VOO. The benefits of rich-phenolic natural OOs on risk factors for chronic diseases have been established in human studies Diet [1]. Within the PREDIMED (Prevención por Dieta Mediterránea) Study we observed a decrease in the atrial fibrillation in cardiovascular disease risk individuals (n = 7440) when the Mediterranean diet was supplemented with extra-VOO [21]. The EUROLIVE Study was a randomized, crossover, controlled study with 200 European healthy volunteers, and three similar OOs but with differences in their phenolic content (from 2.7 to 360 ppm) [22]. From our EUROLIVE results an increase in HDL cholesterol and a decrease in the oxidative damage to lipids, including the *in vivo* oxidized LDL a recognized biomarker for CHD [23], in a dose-dependent manner of the phenolic content of the OO administered were observed. Recently in a subsample of the EUROLIVE study we have reported: 1) an increase of the HDL functionality, evidenced by an increase in the cholesterol efflux from cells, linked to the high-phenolic content OO versus the low content one [24]; and 2) a decrease in total and small LDL particles [25]. Total LDL particles (LDL-P) is now considered to be a biomarker for CHD with the same or higher predictive power than LDL cholesterol [26]. Benefits of high phenolic VOO consumption on other risk factors particularly inflammation, endothelial function and thrombosis markers have also been reported *in vivo* in humans, including a nutrigenomic effect modulating atherosclerosis-related genes towards a protective mode [1, 27]. In front of other edible oils, VOO reduced the postprandial endoplasmic reticulum stress in obese people [28].

In our experience, functional olive oils enriched with phenolic compounds provide more health benefits than the parental VOOs. A functional olive oil enriched with its phenolics (990 ppm), when comparing with the parental VOO (200 ppm of phenolic compounds), was able to reduce the endothelial dysfunction [29] and to enhance the expression of cholesterol efflux related genes in *vivo* in pre- and stage 1 hypertensive patients [30]. Within the VOHF (Virgin Olive oil and HDL Functionality) Study we have recently reported that a functional olive oil enriched with OO phenolics (500 ppm) and with them and those of thyme (500 ppm equimolar) improved the lipoprotein particle subclasses distribution and their associated atherogenic ratios [31, 32]. The functional OOs used in the studies referred to above were enriched from isolated OO phenolic extracts. In the NUTRAOLEOUM study, however, a “coupage” of natural olive varieties together with selected extraction procedures were used for making the OVOO. Besides a more equilibrate micro-nutrient composition, the organoleptic characteristic profile, corresponding to a natural VOO, is expected to enhance the consumers acceptation of the NUTRAOLEOUM OOVO.

The NUTRAOLEUM Study will also evidence for the first time whether the OO triterpenes can provide benefits for human health. Despite a large body of experimental data reporting bioactive effects [4–6], at present, and to the best of our knowledge, no human randomized controlled trial for assessing the *in vivo* potential benefits in humans of oleanolic and maslinic acid from olive oil has been undertaken. We are aimed to take the challenge on the issue in order to provide first level evidence whether triterpenes from olive oil have, or have not, bioactivity *in vivo* in humans. Data on the bioavailability of oleanolic and maslinic acid in humans are scarce. The NUTRAOLEUM Study will provide for the first time information from a randomized controlled trial on the bioavailability and disposition in humans of the oleanolic and maslinic acids present in OOs. Olive oil, a recognized healthy food, can not be consumed in great quantities. Due to this, an increase in the OO bioactive components, such as triterpenes (i.e. FOO) and phenolic compounds (i.e. both FOO and OVOO) is a way for enhancing OO health-promoting properties whilst consuming the same or less fat. Currently, functional foods are developed to improve the properties of natural food components. However, functional foods must be tested in human clinical intervention trials with an appropriate design. Consumers are more and more frequently asking for an “added value” in the nutritional properties of the food to be purchased. Thus, answers from the Olive Oil Industry and from researchers, concerning functional olive oils with tested healthy properties are needed.
